# 

**Published:** 1887-09-24

**Authors:** 


### THE HOSPITAL

An Institution, Family, and Parochial Journal of Hospitals, Asylums, & all Agencies for the Care of the Sick, Criticism & News.

### CONTENTS.

**Sceptical Doctors** - - - - - 423

**Words of Consolation.**—Chapter LI. The Sufferings of Christ - 424

**Safe from Scarlet Fever.** By EDWIN CHADWICK, C.B. - 425

**Amusements for Convalescents.**—Palmistry Notes - 426

**First Days in Africa.** By a MEDICAL MISSIONARY - 427

**St. Mary's and the Working Classes.** By THOMAS RYAN - 428

**Hospital Administration.—Alcohol at Provincial Hospitals** - 429

**Notes and News.**—Miscellaneous Items.—Photographic Views and Albums.—The Hospitals Association Meetings.—The Bridgewater State Procession.—Vacancies.—Sea-water Baths, etc., etc. - 430

**Annotations.**—Honest Yorkshire Farmers.—"The Cow with the Iron Tail."—Wholesale Poisoning - 433

**Kate Mountstephen.** By C. G. FURLEY.—Chap. V. The Hospital on Fire - 434

**Poetry.**—In a Hospital Mortuary. By a HOSPITAL NURSE - 435

**Incidents of Animal Life.**—About Birds.—Mother Partridge's Instinct.—A Musical Parrot - 436

**A Divided House in a Cathedral City** - 436

**The Editor's Letter-box.**—Massage and Medical Electricity - 436

**The Children's Corner.**—Puzzles, Answers, Letters, etc. - 437

**Amusements with Profit** - 437

**In- and Out-Patients' Hospital Diary.**—General and Special Hospitals - 438

